# Clinical features and surgical outcomes of young patients with lung adenocarcinoma manifesting as ground glass opacity

**DOI:** 10.3389/fonc.2022.979522

**Published:** 2022-09-14

**Authors:** Rirong Qu, Dehao Tu, Yixin Cai, Wei Ping, Xiangning Fu

**Affiliations:** ^1^ Department of Thoracic Surgery, Tongji Hospital, Tongji Medical College, Huazhong University of Science and Technology, Wuhan, China; ^2^ Department of Thoracic Surgery, Yueyang Central Hospital, Yueyang, China

**Keywords:** lung adenocarcinoma, ground glass opacity, young patients, clinical features, prognosis

## Abstract

**Background:**

More and more ground glass opacity associated lung adenocarcinoma (GGO-LUAD) have been diagnosed in young patients nowadays. Our study aims to investigate the clinical features and surgical outcomes of young patients with GGO-LUAD.

**Methods:**

Patients aged ≤ 40 years who were diagnosed as lung adenocarcinoma and who underwent video assisted thoracoscopic surgery (VATS) were retrospectively reviewed from January 2017 to December 2018. According to radiological appearance of the patient’s lesions, they were divided into a solid nodule (SN) group and GGO group. The pathological subtypes, surgical procedures and nodules size were analyzed, and the clinical features and prognosis were evaluated between these patients.

**Results:**

A total of 165 patients were included, of which 133 were in the GGO group and 32 in the SN group. Both the GGO group and the SN group had a higher proportion of females and non-smokers. Compared with patients (15.63%) in the SN group, there are more patients (27.8%) under the age of 30 in the GGO group. Pathological findings showed 83.5% of lesions were pre-invasive lesions in the GGO group, although 16.5% of lesions were invasive adenocarcinoma, whereas in the SN group, 96.9% were invasive adenocarcinoma. The GGO group had significantly better histological characteristics and prognosis than the SN group. Perioperative complications occurred in only 6 patients, including pneumonia in one patient, pneumothorax in two patients, and prolonged air leak in three patients. No other serious complications or deaths occurred. After a median follow-up time of 41.2 ± 7.2 months (32-56), the 3-year recurrence free survival (RFS) (100%) and overall survival (OS) (100%) of the GGO group were significantly higher than those (93.42% and 96.88%) in the SN group.

**Conclusions:**

Young patients with GGO-LUAD are mainly non-smokers and female. Most of these patients were early-stage with good prognosis after surgery.

## Introduction

Lung cancer is the most common cause of cancer-related death in China and worldwide (1, 2). Since more than half of lung cancer patients are already in the advanced stage at the time of diagnosis, the current 5-year overall survival is still only about 15% (3). In order to enable more lung cancer patients to be diagnosed and treated at an early stage, low-dose computed tomography (LDCT) has been widely used in the screening of lung cancer in recent years. The result of the US National Lung Screening Trial (NLST) shows that the application of LDCT has reduced the mortality rate of lung cancer by nearly 20% (4). It is through the use of LDCT that many early stage lung cancers with GGO as an imaging feature have been detected (5). Although GGO is not necessarily a manifestation of malignancy, persistent GGO could possibly be a manifestation of lung adenocarcinoma, including adenocarcinoma *in situ* (AIS), minimally invasive adenocarcinoma (MIA), and invasive adenocarcinoma (IAC) (6). Two LDCT screening studies from China found that the majority (74.1-95%) of lung cancers detected by screening were early stage, and 70.4-95.5% of lung cancers were GGO lesions (7).

In the past, we focused more on the diagnosis and treatment of lung cancer in the elderly and young people under the age of 40 are considered non-high-risk population for lung cancer. With the popularity of LDCT and the pandemic of COVID-19 (many young people suspected of having COVID-19 infection also routinely undergo LDCT examination), many ground glass opacities associated lung adenocarcinoma in young patients were incidentally detected on CT scans. Therefore, the diagnosis and treatment of GGO in young patients has become a clinical issue of great concern. Many studies have proved that the prognosis of GGO-LUAD is excellent, the 5-year overall survival (OS) is close to 100% (8, 9), but lack of attention has been paid to the clinical features and prognosis of GGO-LUAD in young patients. In addition, there is no report on the comparison of clinical features and prognosis of young lung adenocarcinoma patients with different radiological appearances.

In the present study, we reviewed the clinical data of patients aged ≤ 40 years who underwent VATS and who were diagnosed as lung adenocarcinoma were reviewed from January 2017 to December 2018. According to radiological appearance of the patient’s lesions, they were divided into a SN group and GGO group. The objective of our study was to evaluate and compare their clinical features and prognosis between two groups.

## Patients and methods

### Patients

This study is a retrospective study. The clinical data of 165 patients aged ≤ 40 years who underwent VATS and who were diagnosed as lung adenocarcinoma were retrospectively analyzed between January 2017 and December 2018. The inclusion criteria were as follows: 1. Single GGO or SN lesion confirmed by thin-slice CT scan and postoperative pathology confirmed lung adenocarcinoma; 2. Age ≤ 40 years; 3. Without other tumors; 4. Mediastinal lymph nodes were evaluated by enhanced CT of the chest before surgery and no mediastinal lymph nodes were significantly enlarged in patients. 5. No distance metastasis; 6. Complete R0 resection. The exclusion criteria were as follows: 1. Postoperative pathology was benign; 2. Mediastinal lymph node metastasis considered on preoperative examination; 3. Postoperative pathologically confirmed non-adenocarcinoma (squamous, large cell, small cell, etc.). Between January 2017 and December 2018, only 10 patients met the other criteria, but the final postoperative pathology was benign (8 with GGO lesions and 2 with SN lesions). According to radiological appearance of the patient’s lesions, they were divided into a SN group and GGO group. The GGO group was further divided into a pure GGO group and a mixed GGO group according to the presence or absence of solid component. Ethics committee of Tongji Medical College of Huazhong University of Science and Technology approved this study and written informed consent was obtained from all patients included in this study. The study was conducted following the Declaration of Helsinki (as revised in 2013).

### Radiological and pathological evaluations

All lesions were evaluated using high-resolution computed tomography (HRCT) images. All chest CT scans were acquired at full inspiration and lesions, particularly GGO nodules, were examined retrospectively. Tumor diameter was defined as the maximum axial diameter of the nodule on the lung window setting, where consolidation was defined as a homogeneous increase in lung parenchymal density that obscured the airway wall and vascular margins, whereas GGO was defined as hazy increased opacity of lung with preserved bronchial and vascular margins. Each lesion on preoperative CT scannings was reviewed blindly by two experienced radiologists.

Pathological subtypes of the tumors were classified according to the International Association for the Study of Lung Cancer (IASLC)/American Thoracic Society (ATS)/European Respiratory Society (ERS) classification of lung adenocarcinomas as AIS, MIA and IAC (10).

### Surgical approach

All patients received single-port VATS. The patient underwent combined intravenous and inhalation general anesthesia with double-lumen endotracheal intubation to maintain single-lung ventilation. Intraoperative rapid frozen section was performed on patients without preoperative pathological diagnosis. Specimens were sectioned at the largest diameter of the tumor for frozen testing. Two levels of each specimen were taken for diagnosis. Two senior pathologists analyzed the frozen sections and reported the results unanimously. When disagreement arose, a third senior pathologist was asked to make the diagnosis. Lobectomy was performed if the intraoperative report suggested that the lesion is invasive adenocarcinoma; sublobar or wedge resection was performed if the intraoperative report suggested that the lesion is pre-invasive tumor. Specific surgical methods and strategies for selecting the extent of resection are described in our previous study (11).

### Follow up

We conduct postoperative follow-up of patients through outpatient or telephone. The follow-up time was calculated from the day after surgery and was followed up until August 2021. Follow-up strategies have been mentioned in previous studies (12). In the first 2 year after surgery, physical examination, tumor markers, abdominal ultrasound and chest CT were reviewed every 6 months routinely; the above indicators were reviewed annually after three years. Bone ECT, brain MRI, or PET-CT was performed only if recurrence or metastasis was suspected.

### Statistical analysis

Measured data were expressed as mean ± standard deviation (SD) and differences between groups were analyzed by t-tests. Counted data were expressed as number or percent, and differences were analyzed using X^2^ or Fisher’s exact tests. Recurrence free survival (RFS) was defined as the time from surgery until first recurrence, or last follow-up. OS was defined as the time from surgery until death from any cause or last follow-up. We used the Kaplan-Meier method to analyze OS and RFS. All statistical analysis was two-sided, and P<0.05 were considered statistically significant. The above data was analyzed using SPSS 23.0 and GraphPad Prism software version 7.0. The above statistical methods were described in our previous study (13).

## Results

### Clinical characteristics of patients and lesions

**Figure 1:**
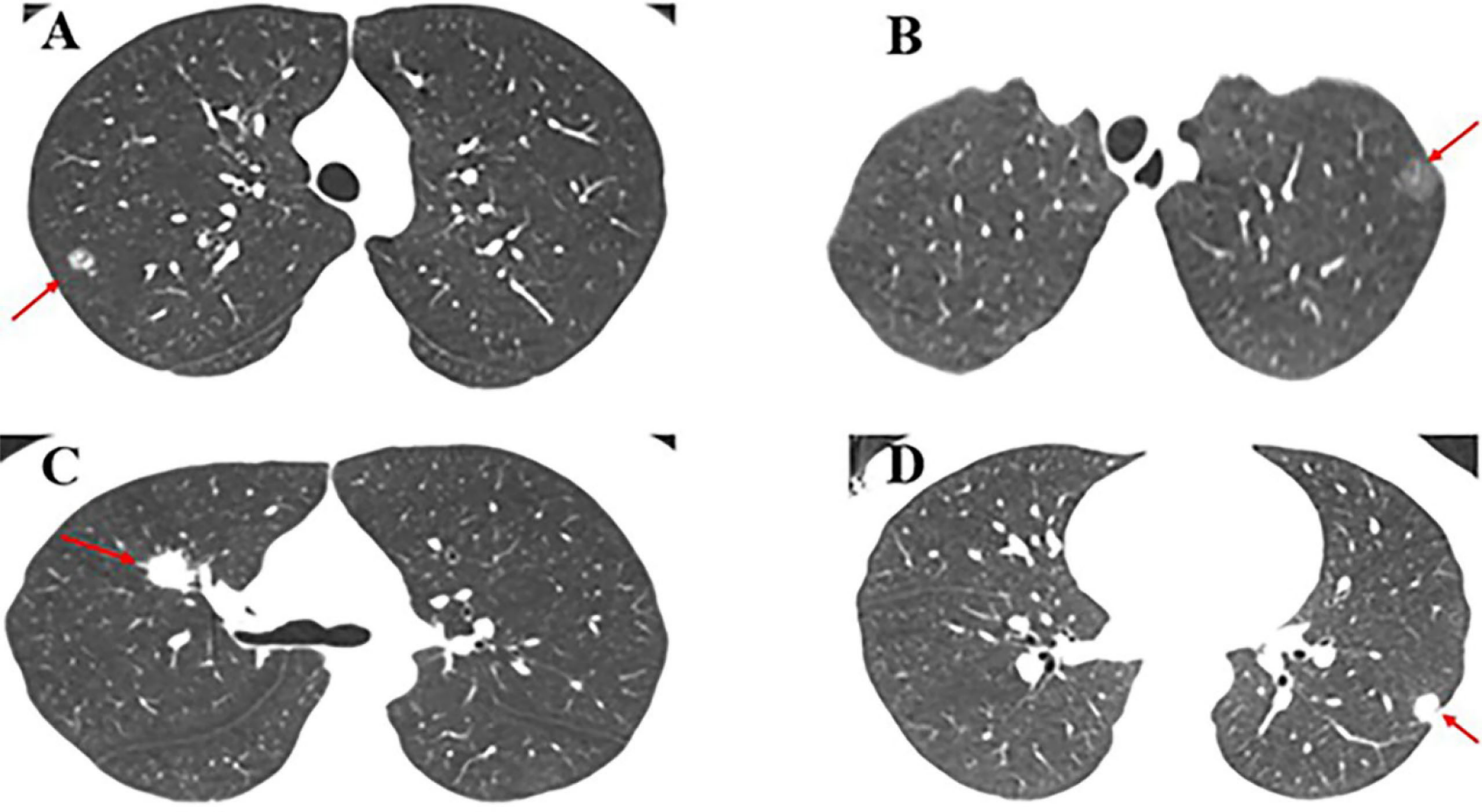
Case presentation. **(A)** A 15-year-old male patient with mGGO (9 mm) in the posterior segment of the right upper lobe underwent single-port VATS segmentectomy. The final pathology was AIS; **(B)** A 17-year-old female patient with pGGO (10 mm) in the posterior segment of the left upper lobe underwent single-port VATS segmentectomy. The final pathology was MIA; **(C)** A 28-year-old female patient with SN (18 mm) in the right upper lobe underwent single-port VATS lobectomy. The final pathology was invasive mucinous adenocarcinoma; **(D)** A 35-year-old female patient with SN (12 mm) in the left lower lobe underwent single-port VATS lobectomy. The final pathology was lepidic predominant adenocarcinoma.

A total of 165 patients were included (117 females and 48 males, with an average age of 34.11 ± 4.36, range 15 to 40 years). Among the 165 lesions, 133 were GGO lesions (74 for pGGO and 59 for mGGO), and 32 were SN lesions. Some cases had GGO and SN on CT (**Figure 1**). The average largest diameter of SN lesions was 22.47 ± 8.71 mm, followed by mGGO lesions (13.16 ± 5.84 mm), and pGGO lesions (9.03 ± 1.93 mm). There were significant differences in the average diameter of the three types of lesions (P<0.001). Among all patients, only ten male patients had former or current smoking history. Twenty-three patients had a family history of tumors, 11 of whom had a family history of lung cancer. The median surveillance time frame before surgery was 11 months (ranging from 1 day to 36.5 months). 86% of the lesions were detected on chest CT during physical examination. The patient characteristics is shown in **Table 1**.

**Table 1:** Clinical features of Patient.

Variables	pGGO (n=74), n (%)	mGGO (n=59), n (%)	SN (n=32), n (%)	P value
Age (Year ± SD)	34.15 ± 4.26	32.96 ± 4.60	35.93 ± 3.47	0.130
≤ 30	17 (22.97)	20 (33.9)	5 (15.63)	
> 30	57 (77.03)	39 (66.1)	27 (84.38)	
Sex				0.389
Male	19 (25.68)	21 (35.59)	8 (25)	
Female	55 (74.32)	38 (64.41)	24 (75)	
Smoking status				0.476
No	71 (95.95)	55 (93.22)	29 (90.63)	
Current or former	3 (4.05)	4 (6.78)	3 (9.38)	
Family history				0.377
Lung caner	5 (6.76)	6 (10.17)	0 (0)	
Other cancer	7 (9.46)	3 (5.08)	2 (6.25)	
No	62 (83.78)	50 (84.75)	30 (93.75)	
Tumor size (mean ± SD, mm)	9.03 ± 1.93	13.16 ± 5.84	22.47 ± 8.71	0.000
Time of lesion first found*				0.183
≤1month	29 (39.19)	25 (42.37)	22 (68.75)	
>1month, ≤6 months	27 (36.49)	23 (38.98)	6 (18.75)	
>6 months, ≤1year	14 (18.92)	8 (13.56)	3 (9.38)	
>1year	4 (5.41)	3 (5.08)	1 (3.13)	
Detection of lesion				0.294
Symptom	3 (4.05)	2 (3.39)	4 (12.5)	
Screening tests	66 (89.19)	52 (88.14)	24 (75)	
Incidental	5 (6.76)	5 (8.47)	4 (12.5)	

*The time from the first discovery of the lesion to the operation; pGGO, pure ground glass opacity; mGGO, mixed ground glass opacity; SN, solid nodule.

Among mGGO lesions, there were 37 lesions with a consolidation-to-tumor ratio (CTR) value of ≤0.5, and 22 lesions with a CTR value of >0.5. The diameter of GGO lesions was more distributed in the range of ≤10mm, accounting for 75.2%; while the diameter of SN lesions was more >10mm, of which ≥20mm lesions accounted for 43.7%. Regardless of whether it is GGO or SN, the distribution of lesions is mainly peripheral, especially GGO lesions (93.9%); in their specific lobe location, both upper lungs were predominant. During the period from the patient’s first discovery of the lesion to the operation, only 12 patients had an increase in lesions, including 5 lesions with an increase in diameter and 7 lesions with a solid component increase. **Table 2** lists the clinical characteristics of lesions.

**Table 2:** Clinical characteristics of Lesion.

Variables	GGO (n=133), n (%)	SN (n=32), n(%)
Radiological appearance		
pGGO	74 (55.6)	-
mGGO	59 (44.4)	-
CTR		
> 0, ≤ 0.5	37 (27.8)	-
> 0.5, < 1	22 (16.5)	-
Tumor size (mean ± SD, mm)	11.1 ± 4.8	22.47 ± 8.7
≤ 10	100 (75.2)	2 (6.3)
> 10, < 20	29 (21.8)	16 (50.0))
≥ 20	4 (3.0)	14 (43.7)
Tumor location		
Central	8 (6.1)	7 (21.9)
Peripheral	125 (93.9)	25 (78.1)
Occupying lobe		
RUL	38 (28.6)	8 (25)
RML	6 (4.5)	4 (12.5)
RLL	25 (18.8)	7 (21.9)
LUL	37 (27.8)	7 (21.9)
LLL	27 (20.3)	6 (18.7)
Growth after first found		
Yes	8 (6.1)	4 (12.5)
No	125 (93.9)	28 (87.5)
EGFR status		
Mutation	22 (16.5)	10 (31.2)
WT	33 (24.8)	8 (25.0)
Not detected	78 (58.7)	14 (43.8)

CTR, consolidation-to-tumor ratio; SN, solid nodule; mGGO, mixed ground glass opacity; pGGO, pure ground glass opacity; RLL, right lower lobe; LUL, left upper lobe; LLL, left lower lobe; RUL, right upper lobe; RML, right middle lobe; EGFR, epidermal growth factor receptor; WT, wild type.

### Pathological characteristics

Among the GGO group, the vast majority were pre-invasive adenocarcinoma and minimally invasive adenocarcinomas (66 AIS and 45 MIA), but 16.5% of the lesions are still invasive adenocarcinoma; among the SN group, 96.9% are invasive adenocarcinoma, and only one lesion is MIA. Compared to the GGO group, the SN group displayed significantly worse pathological subtypes (P<0.001), more visceral pleural invasion (0.8% vs. 12.5%, P<0.001), more lymphovascular invasion (0% vs. 12.5%, P<0.001), more spread through air spaces (0.8% vs. 25%, P<0.001) and more lymph node metastasis (0% vs. 18.8%, P<0.001). The six patients with lymph node metastasis were all in the SN group. The postoperative pathological results of tumors are described in **Table 3**.

**Table 3:** Pathological analysis of Lesion.

Characteristic	pGGO (n=74), n (%)	mGGO (n=59), n (%)	SN (n=32), n (%)	P value
Pathology				0.000
AIS	47 (63.5)	19 (32.2)	0 (0)	
MIA	22 (29.7)	23 (38.9)	1 (3.1)	
IAC	5 (6.8)	17 (28.9)	31 (96.9)	
Predominant subtype				0.00
Acinar	3 (4.0)	10 (13.5)	16 (50)	
Lepidic	1 (1.4)	3 (8.6)	4 (12.5)	
Papillary	1 (1.4)	4 (6.8)	8 (25)	
Solid	0 (0)	0 (0)	1 (3.1)	
Mucinous	0 (0)	0 (0)	2 (6.3)	
VPI	0 (0)	1 (1.7)	4 (12.5)	0.005
LVI	0 (0)	0 (0)	1 (3.1)	0.194
STAS	0 (0)	1 (1.7)	8 (25)	0.000
Pathological N1/N2	0 (0)	0 (0)	6 (18.8)	0.000

SN, solid nodule; mGGO, mixed ground glass opacity; pGGO, pure ground glass opacity; IAC, invasive adenocarcinoma; MIA, micro invasive adenocarcinoma; AIS, adenocarcinoma in situ; VPI, visceral pleural invasion; LVI, lymphovascular invasion; STAS, spread through air spaces.

### Perioperative results and complications

Of the 133 GGO lesions, the main surgical procedure was sublobar resection, 85 patients underwent segmentectomy, 25 patients underwent wedge resection, 23 patients underwent lobectomy, of which 18 patients were mGGO lesions. Among the 32 SN lesions, the main surgical procedure was lobectomy, and only 3 patients underwent segmentectomy. There was no difference in the postoperative hospital stay and postoperative chest tube duration among the two groups. A total of 6 patients had postoperative complications, including two with pneumothorax, three with prolonged air leak, and one with pneumonia. No other serious complications or deaths occurred, and all patients were discharged from the hospital smoothly. A detailed description of the perioperative results and complications are provided in **Table 4**.

**Table 4:** Perioperative results and complications.

Variables	pGGO (n=74), n (%)	mGGO (n=59), n (%)	SN (N=32), n (%)
Surgical procedure			
Wedge resection	16 (21.6)	9 (15.3)	0 (0)
Segmentectomy	53 (71.6)	32 (54.2)	3 (9.4)
Simple	37 (50.0)	25 (42.3)	1 (3.1)
Complex	16 (21.6)	7 (11.9)	2 (6.3)
Lobectomy	5 (6.7)	18 (30.5)	29 (90.6)
Postoperative chest tube duration (day)	2.22 ± 0.56	2.21 ± 0.59	2.56 ± 0.62
Postoperative hospital stay (day)	3.46 ± 0.75	3.48 ± 0.85	3.56 ± 0.58
Perioperative complications			
Pneumothorax	1 (1.4)	1 (1.7)	0 (0)
Pneumonia	0 (0)	0 (0)	1 (3.1)
Prolonged air leak	1 (1.4)	1 (1.7)	1 (3.1)

SN, solid nodule; mGGO, mixed ground glass opacity; pGGO, pure ground glass opacity.

### Survival analysis

**Figure 2:**
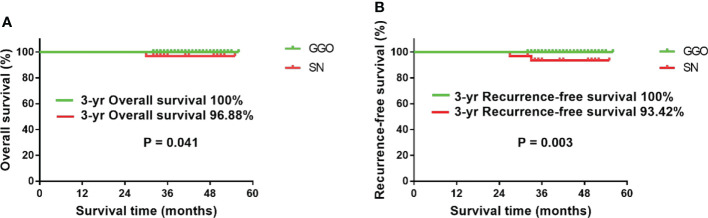
Survival analysis of the two groups of patients. **(A)** The 3-year OS of the GGO group was significantly better than that of the SN group (P=0.041); **(B)** the 3-year RFS of the GGO group was significantly better than that of the SN group (P=0.003).

Until August 2021, all 165 patients were successfully followed up, with an average follow-up time of 41.2 ± 7.2 months (32–56). A total of 2 patients developed postoperative recurrence, and one patient died due to multiple distant metastases. All three of these patients were in the SN group and had lymph node metastases on postoperative pathology. The results of the survival analysis showed that the 3-year RFS and OS of the GGO group were 100%, while the 3-year RFS and OS of the SN group were 93.42% and 96.88%, respectively (**Figure 2**).

## Discussion

In the past decade, with the advancement of medical technology and the improvement of treatment methods, the incidence and mortality of lung cancer have declined. But lung cancer remains the leading cause of cancer-related deaths worldwide (1). Generally, it is rare for people under 40 to suffer from lung cancer, and the incidence is about 0.6%-4.6% (14–17). However, with the implementation of lung cancer screening (LCS) and the wide application of LDCT, more and more early-stage lung adenocarcinomas with GGO as radiological appearances have been diagnosed. Among them are young patients whose clinical features and prognosis are still unclear. This study collected the clinical data of 165 young patients who underwent VATS in our hospital between January 2017 and December 2018 and were pathologically diagnosed as lung adenocarcinoma, of which 133 patients’ lesions were GGO, and 32 patients’ lesions were SN. We found that both the GGO group and the SN group are mainly female and non-smokers, which is consistent with the findings of previous studies (15, 18). It is worth noting that in our study, 23 patients had a family history of tumors, 11 of whom had a family history of lung cancer, while in Wu’s study (19), none of the 12 teenagers with lung adenocarcinoma had a family history of lung cancer. This may be related to the small sample size, but it also reminds us that for young patients with a family history of lung cancer, their risk of developing lung cancer may increase.

The causes of lung cancer in young women and non-smokers remain unclear. Tobacco is the leading cause of lung cancer in adult patients (20). In this study, only 10 patients had a history of smoking and were all male. So how do non-smoking female patients get lung cancer? It has been reported that patients with a history of long-term exposure also have an increased risk of developing lung cancer, such as exposure to second-hand smoke, air dust pollution, and kitchen fumes (21, 22). However, there are two young patients under the age of 18 in this study. They have neither smoking history nor above exposure history. Exactly what causes them to develop lung cancer remains to be studied. A recent study showed that compared with elderly patients, young patients with lung adenocarcinoma have different driver mutation genes (23). In order to further understand the mechanism of young patients with GGO-LUAD, more relevant research is urgently needed in the future.

GGO’s management strategy has not yet reached a consensus at home and abroad. In the current study, we chose the surgical procedure mainly through comprehensive consideration of the nature of the lesion, the size of the lesion, the location of the lesion, the results of rapid frozen section, and the cardiopulmonary function of the patient. After a median follow-up of 41.2 ± 7.2 months, the 3-year RFS and OS of the GGO group were 100%, while the 3-year RFS and OS of the SN group were 93.42% and 96.88%, respectively. This is consistent with the previous findings (12, 24). For young patients with GGO, sublobar resection should be the first choice. Although lobectomy is still the best surgical approach for early stage lung cancer, more and more studies have shown that GGO-LUAD has an excellent prognosis after sublobar resection, and the 5-year OS is close to 100% (25, 26). Therefore, sublobar resection may be the best surgical approach for such patients.

Since AIS has an excellent prognosis, whether they should be surgically removed or followed-up remains controversial. Some people believe that AIS is an extremely indolent disease that should be followed up rather than surgically removed. According to Fleischner Society guidelines (27), patients with GGO should receive a CT scan every 1-2 years for 5 years. Surgical resection should be considered only if nodule size or solid component has progressed. However, GGO could progress even when smaller than 6 mm and stable for 5 years (28). This means that some stable GGO lesions still require follow-up after 5 years, but as the number of CT scans increases during follow-up, so does the radiation exposure. Research suggests that frequent follow-up CT scans may harm patients (29). For patients with AIS, the radiation exposure is greatly reduced from follow-up CT scans after surgical resection. Another reason why AIS should be removed early is that if the GGO increases in diameter or the solid component increases during follow-up, the extent of surgical resection may be expanded and the patient’s prognosis may be worse (30).

The lung cancer screening strategy for young patients may be different. The benefits of LCS increase as the risk of lung cancer increases. Therefore, the current LCS guidelines issued by countries around the world are mainly aimed at people at high risk of lung cancer. However, the age of the screening population is not completely consistent. The latest NCCN guidelines suggest that 55-77 years old or older who are at greater risk of lung cancer should be screened (31). The International Early Lung Cancer Action Plan recommends LCS for people over 40 years old (32). China’s latest LCS guidelines recommend that people aged 45-75 should be screened (33), because cancer statistics from China show (2) that the age-specific incidence and mortality of lung cancer have increased significantly after 45 years. For screening frequency, young patients with GGO should receive a CT scan every 1-2 years for 5 years or a surgical resection if nodule size or solid component has progressed, according to Fleischner Society guidelines (27). The youngest patient in this study was 15 years old, and there were nine patients ≤25 years old. In the Wu’s study (19), the youngest patient was 14 years old, and all patients are younger than 20. This may indicate that GGO-LUAD has a younger trend, so the age of screening for this population may be advanced.

Since GGO-LUAD is generally considered as an indolent disease, overdiagnosis and overtreatment is a major concern. To reduce overdiagnosis and overtreatment, it is essential to make sure that benign lesions are not treated as malignancies, and GGO-LUAD are not treated the same way as the solid tumors. However, the best way to solve this problem is to monitor its changes within a certain period of time. According to Fleischner Society Guidelines (27), GGOs <6mm do not require follow-up, and ≥6mm GGOs are confirmed persistence within the period of 6-12 months, then CT will continue to be reviewed every 1-2 years for 5 years; if the solid component or size of the lesion during the period increase, then directly consider surgical resection. The latest NCCN guidelines also show that pGGO < 6mm does not require monitoring (34), while pGGO and mGGO ≥ 6mm have different monitoring strategies. For pGGO ≥ 6 mm if it has no any change during 6-12 months, CT will be reviewed every 2 years for 5 years; for mGGO ≥ 6mm if it has no any change during 6-12 months, CT will be reviewed annually for 5 years. Within 3-6 months of follow-up, most benign lesions will disappear or shrink, and for lesions that have not undergone any changes, 92.6% may be malignant (35). Since there is still no effective method to predict the growth of GGO, the specific intervention time for GGO remains unclear. Previous studies have suggested that patient age and nodule location should be considered when deciding on aggressive surgical intervention for persistent small GGO lesions (5). Early intervention is recommended for younger patients with peripheral GGO lesions that can be completely resected by sublobar resection. Follow-up is recommended for elderly patients who have a short life expectancy or small central GGO lesions which require lobectomy. Therefore, except for centrally located GGO lesions, for the persistent GGO, surgical resection should be advocated as soon as possible. However, the specific surgical method must be comprehensively considered according to the location of the lesion and the age of the patient.

There are several shortcomings of this study that must be considered. First, the nature of this retrospective study does not avoid the existence of intrinsic biases and We did not analyze patients with similar pulmonary nodules who did not undergo surgery. Second, this study is a single-institution study and the sample size is not large enough, and a multi-center study with a large sample is still needed for later validation. Third, the clinical characteristics of young patients with lung cancer will vary between different races (14) and our study is only for the Chinese population. Fourth, the follow-up period of this study is still not long enough and longer follow-up data is needed to better assess the prognosis of patients.

In summary, GGO-LUAD in young patients is mainly female and non-smokers. Compared to the SN group, they have significantly better histological characteristics and prognosis after surgery. Further research into the molecular mechanisms underlying the development of GGO-LUAD in young patients is needed.

## Data availability statement

The original contributions presented in the study are included in the article/supplementary material. Further inquiries can be directed to the corresponding author.

## Ethics statement

The studies involving human participants were reviewed and approved by Tongji Medical College of Huazhong University of Science and Technology. Written informed consent to participate in this study was provided by the participants’ legal guardian/next of kin.

## Author contributions

RQ, YC, WP, and XF contributed to the design of the study and the performing of the procedure. RQ and DT acquired and analyzed the data. RQ drafted the manuscript. RQ, WP, and XF revised and edited the manuscript. All authors contributed to the article and approved the submitted version.

## Funding

This work was funded by the National Natural Science Foundation (No. 81902347 to WP) and the Tongji Hospital Clinical Research Flagship Program (No. 2019CR107 to XF).

## Previous publication statement

The abstract of the manuscript has been accepted as E-Poster presentation for the 2022 World Conference on Lung Cancer (WCLC) numbered EP01.03-11.

## Conflict of interest

The authors declare that the research was conducted in the absence of any commercial or financial relationships that could be construed as a potential conflict of interest.

## Publisher’s note

All claims expressed in this article are solely those of the authors and do not necessarily represent those of their affiliated organizations, or those of the publisher, the editors and the reviewers. Any product that may be evaluated in this article, or claim that may be made by its manufacturer, is not guaranteed or endorsed by the publisher.
